# Functional Characterization of Two *β-Hexosaminidase A* Isoforms During Ovarian Development in *Macrobrachium nipponense*

**DOI:** 10.3390/ijms26125459

**Published:** 2025-06-06

**Authors:** Zhiming Wang, Sufei Jiang, Wenyi Zhang, Shubo Jin, Yiwei Xiong, Ming Xu, Zijian Gao, Mingjia Xu, Hui Qiao, Hongtuo Fu

**Affiliations:** 1 National Demonstration Center for Experimental Fisheries Science Education, Shanghai Ocean University, Shanghai 201306, China; 2 Key Laboratory of Freshwater Fisheries and Germplasm Resources Utilization, Ministry of Agriculture and Rural Affairs, Freshwater Fisheries Research Center, Chinese Academy of Fishery Sciences, Wuxi 214081, China; 3 Wuxi Fisheries College, Nanjing Agricultural University, Wuxi 214081, China

**Keywords:** *Macrobrachium nipponense*, *β-hexosaminidase A*, ovarian maturation, RNAi

## Abstract

In this study, we identified two *β-hexosaminidase A* isoforms (*Mn-HexA1* and *Mn-HexA2*) in *Macrobrachium nipponense* through bioinformatics analysis and characterized their phylogenetic relationships. The open reading frames of *Mn-HexA1* and *Mn-HexA2* were 1641 bp (encoding 546 amino acids) and 1473 bp (encoding 490 amino acids), respectively. Both isoforms exhibited high conservation, sharing five identical functional domains, with 58.43% amino acid sequence similarity. Quantitative PCR analysis revealed that *Mn-HexA1* expression was significantly higher than *Mn-HexA2* across all developmental stages and tissues. During embryonic development, *Mn-HexA1* showed elevated expression at the ZS, L15, and PL10, while *Mn-HexA2* was upregulated only at L15 and PL10. In the breeding season and non-breeding season, *Mn-HexA1* and *Mn-HexA2* were predominantly expressed in the hepatopancreas at levels significantly higher than in other tissues. Moreover, their expression in most tissues was higher during the breeding season than in the non-breeding season. *RNA interference* experiments revealed that knockdown of both *Mn-HexA* isoforms significantly accelerated ovarian development in *M. nipponense*, with the *Mn-HexA1*-silenced group exhibiting faster progression than the *Mn-HexA2*-silenced group. These results demonstrate that *Mn-HexA* genes function as negative regulators of ovarian maturation, with *Mn-HexA1* exerting a stronger inhibitory effect than *Mn-HexA2*.

## 1. Introduction

*Macrobrachium nipponense* is a commercially important freshwater crustacean species widely cultivated in Southeast Asia. As the only indigenous freshwater shrimp species that has achieved large-scale aquaculture production in China, *M. nipponense* plays a vital role in the regional aquaculture industry. According to recent statistics, the annual production of *M. nipponense* reached 226,392 tons in 2023 [1,2], demonstrating its significant economic value. The reproductive biology of *M. nipponense* presents unique challenges for aquaculture production. During the breeding season from April to October, female prawns exhibit accelerated gonadal maturation and shortened reproductive cycles. This reproductive strategy allows multiple generations to coexist in the same culture pond, which leads to a decline in the growth rate, resistance, and survival rate of shrimp seedlings, as well as increased risk of hypoxia due to higher stocking densities. These factors collectively contribute to substantial economic losses in *M. nipponense* aquaculture operations [3,4]. However, the molecular mechanism behind the precocious puberty of *M. nipponense* is still poorly understood, so it is of great significance to carry out relevant research.

The hepatopancreas has been well documented to play a pivotal role in ovarian maturation processes across various crustacean species [5]. As the primary metabolic organ, it facilitates nutrient digestion and absorption, subsequently transporting these essential dietary components to developing ovaries through hemolymph during oogenesis [6]. Building upon these established findings, our research team conducted a comprehensive transcriptomic comparison of hepatopancreatic tissue from female *M. nipponense* across ovarian developmental stages O1 to O5. Intriguingly, our analysis revealed two *β-hexosaminidase A* homologs (*Mn-HexA1* and *Mn-HexA2*) that exhibited significant upregulation during the transition from stage O1 to O2 hepatopancreas [7]. Further bioinformatic investigation through KEGG pathway analysis identified substantial enrichment of the lysosome signaling pathway—a pathway demonstrating strong correlation with ovarian development [8]. Notably, *Mn-HexA* genes were prominently represented within this pathway. These collective findings strongly suggest that *Mn-HexA1* and *Mn-HexA2* may serve as functional regulators in the ovarian development of *M. nipponense*.

Hexosaminidases, members of the glycoside hydrolase (GH) 20 family, are primarily responsible for hydrolyzing terminal GalNAc residues from GM2 gangliosides within lysosomes. Reproductive studies have demonstrated remarkable hexosaminidase activity in spermatozoa across diverse species, including humans [9], canines [10], and insects [11]. It is involved in the combination and penetration with sperm–zona pellucida in vitro, which is very important for egg–sperm recognition [12,13]. Moreover, hexosaminidase has been shown to play an important role in primary binding between gametes in multiple species, including humans [14], hamsters [15], and *Phallusia mammillata* [16], in addition to modifying the sperm receptor glycoprotein on the egg envelope to block polyspermy. Notably, the regulatory function of hexosaminidase on the development of biological ovaries has not yet been studied, and there is also almost no research on it in crustaceans.

Building upon these findings, we conducted comprehensive bioinformatics analyses to characterize the sequence features and phylogenetic relationships of *Mn-HexA1* and *Mn-HexA2* in *M. nipponense*. Using quantitative real-time PCR (qPCR), we systematically investigated the expression profiles of these two isoforms across (1) different tissues during the breeding season and non-breeding season, (2) various embryonic developmental stages, (3) distinct ovarian developmental phases, and (4) the hepatopancreas at different maturation stages. The spatial distribution patterns of *Mn-HexA1* and *Mn-HexA2* transcripts in ovarian and hepatopancreatic tissues were further examined through in situ *hybridization* (ISH). To functionally validate their regulatory roles, *RNA interference* (RNAi) technology was employed to specifically knock down these genes and assess their impacts on ovarian development. This integrative approach provides novel insights into the molecular mechanisms underlying female precocious maturation in *M. nipponense*, potentially offering solutions to this persistent challenge in aquaculture practice.

## 2. Results

### 2.1. Full-Length Sequence Analysis of Mn-HexA1 and Mn-HexA2

Molecular characterization revealed that the *HexA1* in *M. nipponense* contains an open reading frame (ORF) of 1641 bp, encoding a 546-amino-acid protein, which we designated as *Mn-HexA1* (GenBank accession no. PV585537). The complete cDNA sequences and deduced amino acid sequences of *Mn-HexA1* are presented in Figure S1. Bioinformatic analysis revealed that the *Mn-HexA1* protein has a predicted molecular weight (Mw) of 62,136.46 Da and a theoretical isoelectric point (pI) of 5.28. Compositional analysis of the full-length *Mn-HexA1* amino acid sequence demonstrated that L-leucine (L) was the most abundant residue (8.2%). Among all residues, the protein contains 51 positively charged amino acids (Arg + Lys) and 68 negatively charged amino acids (Asp + Glu), with a predicted molecular formula of C_2813_H_4254_N_724_O_824_S_23_. Secondary structure prediction indicated that *Mn-HexA1* comprises 16 α-helices, 3 η-helices, 18 β-sheets, 1 α-turn, and 12 β-turns, as illustrated in Figure S2. Furthermore, a signal peptide (Sec/SPI type) was identified at positions 1–20 bp (amino acid sequence: MLGTKLLLVFVTVAVGPAIA).

Similarly, the *HexA2* in *M. nipponense* was found to possess an ORF of 1473 bp, encoding a 490-amino-acid polypeptide, which we designated as *Mn-HexA2* (GenBank accession no. PV585538). The complete cDNA sequences and deduced amino acid sequences of *Mn-HexA2* are presented in Figure S1. The *Mn-HexA2* protein was predicted to have a molecular weight (Mw) of 56,902.10 Da with a theoretical isoelectric point (pI) of 4.94. Amino acid composition analysis revealed that L-leucine (L) was the most abundant residue (8.8%) in the full-length *Mn-HexA2* sequence. The protein contains 46 positively charged residues (Arg + Lys) and 73 negatively charged residues (Asp + Glu), with a predicted molecular formula of C_2574_H_3858_N_658_O_765_S_20_. Secondary structure prediction demonstrated that *Mn-HexA2* consists of 13 α-helices, 17 β-sheets, and 12 β-turns, as illustrated in Figure S2. Additionally, a Sec/SPI-type signal peptide was identified at positions 1–17 bp, corresponding to the N-terminal amino acid sequence MLVLLVTLSMAVNLSAG.

Sequence analysis revealed that *Mn-HexA1* contains two conserved domains: Glycohydro_20b2 (positions 32–156) and GH20_HexA_HexB-like (positions 182–529). Similarly, *Mn-HexA2* possesses two conserved domains: Glycohydro_20b2 (positions 29–182) and GH20_hexosaminidase superfamily (positions 203–481), demonstrating high sequence similarity between *Mn-HexA1* and *Mn-HexA2*. Notably, both isoforms share five identical functional domains: (1) β-hexosaminidase A; (2) β-hexosaminidase A, eukaryotic type, N-terminal; (3) β-hexosaminidase A-like, domain 2; (4) glycoside hydrolase family 20, catalytic domain; and (5) glycoside hydrolase superfamily. Subcellular localization predictions indicated that residues 21–546 of *Mn-HexA1* and 21–490 of *Mn-HexA2* correspond to non-cytoplasmic domains. Furthermore, transmembrane domain analysis confirmed the absence of any transmembrane regions in either *Mn-HexA1* or *Mn-HexA2.*

### 2.2. Similarity Comparison and Phylogenetic Analysis

**Figure 1:**
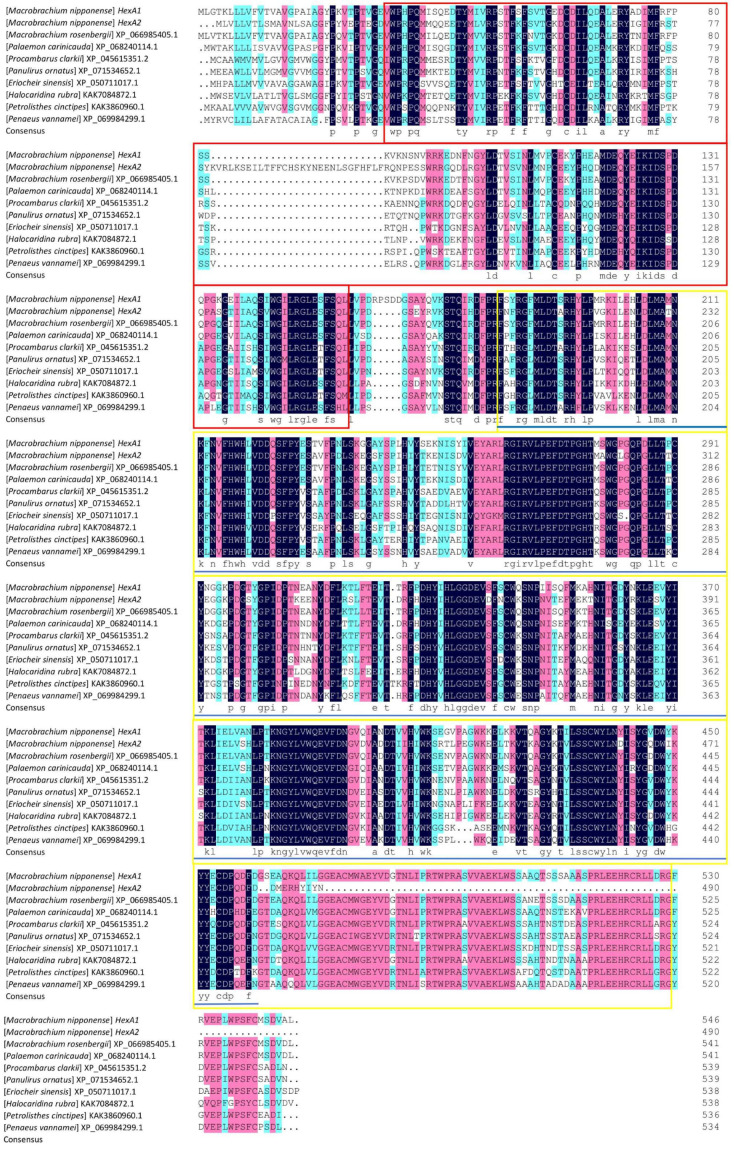
Multiple-sequence alignment of amino acid sequences across various species. Conserved regions are highlighted in black, while conservative substitutions are indicated in pink. The red box denotes the Glycohydro_20b2 conserved domain in both *Mn-HexA1* and *Mn-HexA2*, the yellow box marks the GH20_HexA_HexB-like conserved domain specific to *Mn-HexA1*, and the blue solid line delineates the GH20_hexosaminidase superfamily conserved domain unique to *Mn-HexA2*.

Sequence alignment analysis using DNAMAN 9.0 revealed 58.43% similarity between *Mn-HexA1* and *Mn-HexA2.* Comparative multiple sequence alignment with homologous genes from other species (listed in phylogenetic order: *Macrobrachium rosenbergii*, *Palaemon carinicauda*, *Procambarus clarkii*, *Panulirus ornatus*, *Eriocheir sinensis*, *Halocaridina rubra*, *Petrolisthes cinctipes*, and *Penaeus vannamei*) demonstrated that *Mn-HexA1* exhibited sequence similarities of 91.76%, 81.17%, 73.57%, 71.56%, 70.56%, 70.45%, 69.26%, and 69.26%, respectively. Correspondingly, *Mn-HexA2* showed sequence similarities of 83.40%, 71.40%, 63.68%, 63.71%, 62.84%, 64.02%, 61.29%, and 64.30% with these same species (Figure 1).

**Figure 2:**
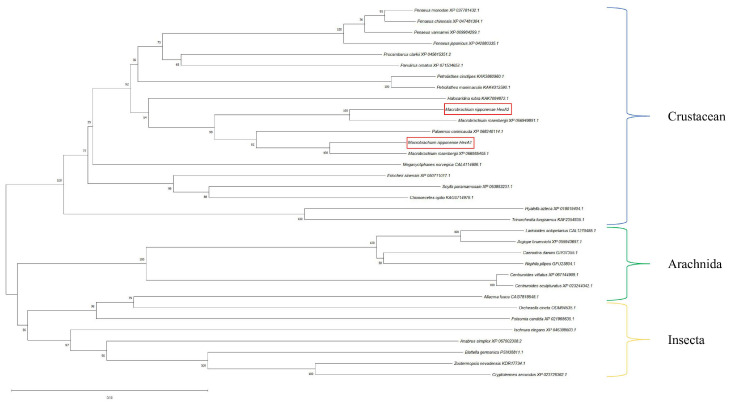
Phylogenetic tree analysis of *Mn-HexA1* and *Mn-HexA2*. The numbers on the branch represent the bootstrap percentages of the phylogenetic tree. Bootstrap copy to 1000. The terminal numbers correspond to GenBank accession numbers. The target genes is marked with the red boxes.

Phylogenetic analysis revealed limited research background for these genes, with homologous sequences from other species in NCBI being predominantly predicted genes. Using MEGA 11.0, we constructed a phylogenetic tree based on the amino acid sequences of *Mn-HexA1*, *Mn-HexA2*, and their homologs from other species. The phylogenetic tree demonstrated that *Mn-HexA1* and *Mn-HexA2* did not cluster with each other initially. Instead, each first grouped with distinct genes from *Macrobrachium rosenbergii*, then with *Palaemon carinicauda* from the same family Palaemonidae, before finally clustering together. Subsequently, they collectively branched toward other crustaceans, including *Halocaridina rubra*, *Penaeus vannamei*, and *Eriocheir sinensis*, ultimately converging with Insecta and Arachnida lineages (Figure 2).

### 2.3. Expression Analysis of Mn-HexA1 and Mn-HexA2

#### 2.3.1. Expression Analysis of *Mn-HexA1* and *Mn-HexA2* at Different Developmental Stages

**Figure 3:**
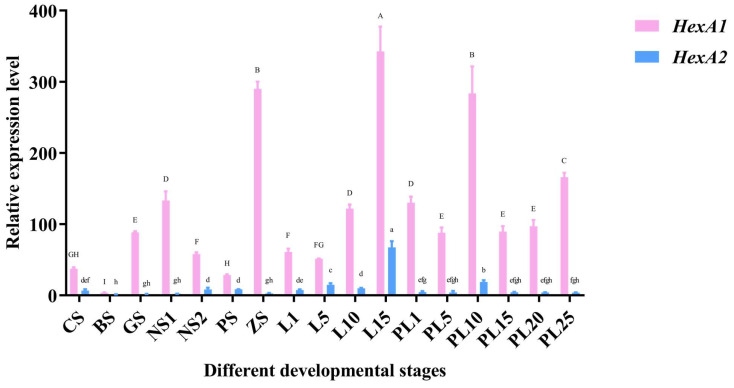
Quantitative analysis of *Mn-HexA1* and *Mn-HexA2* expression patterns during various developmental stages by qPCR. Data are presented as mean ± SD (n = 6). Extremely significant differential expression was observed between *Mn-HexA1* and *Mn-HexA2* across all developmental stages (*p* < 0.01). Uppercase letters indicate statistically significant differences among *Mn-HexA1* expression groups (*p* < 0.05), while lowercase letters denote significant differences among *Mn-HexA2* expression groups (*p* < 0.05). Developmental stages from CS to PL25 are detailed in Table S1.

The embryonic and developmental expression profiles of *Mn-HexA1* and *Mn-HexA2* are presented in Figure 3. During early embryonic development, *Mn-HexA1* exhibited peak expression at the ZS (zoea stage), whereas *Mn-HexA2* showed maximal expression during the PS (protozoea stage). In post-embryonic development, both isoforms demonstrated their highest expression levels at L15 (15-day-old larvae after hatching from the embryonic membrane). Throughout the planktonic larval stages, PL10 (the 10th day after metamorphosis) represented the developmental peak for both genes. Comparative analysis revealed that L15 was the overall stage of maximal expression for both *Mn-HexA1* and *Mn-HexA2*. Importantly, *Mn-HexA1* expression levels were significantly higher than *Mn-HexA2* across all developmental stages (*p* < 0.01).

#### 2.3.2. Expression Analysis of *Mn-HexA1* and *Mn-HexA2* in Different Tissue

**Figure 4:**
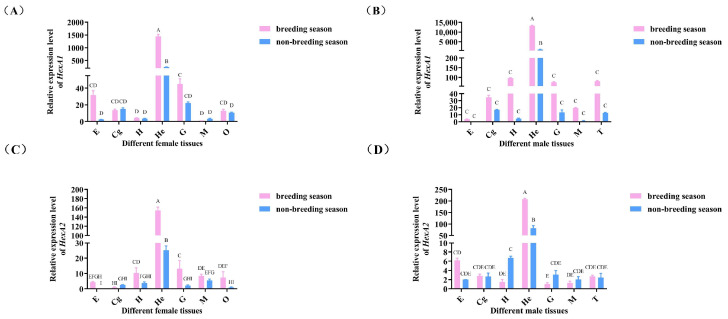
Tissue expression analysis of (**A**) *Mn-HexA1* in females, (**B**) *Mn-HexA1* in males, (**C**) *Mn-HexA2* in females, and (**D**) *Mn-HexA2* in males during breeding season and non-breeding season quantified by qPCR. E: eyestalk; Cg: cerebral ganglion; H: heart; He: hepatopancreas; G: gill; M: muscle; O: ovary; T: testis. Data are presented as mean ± SD (n = 6). Different superscript letters indicate statistically significant differences (*p* < 0.05).

As shown in Figure 4, both *Mn-HexA1* and *Mn-HexA2* exhibited predominant expression in the hepatopancreas, with significantly higher levels compared to other examined tissues (*p* < 0.01). Notably, gene expression in various tissues of both female and male prawns was generally elevated during the breeding season relative to the non-breeding season.

#### 2.3.3. Expression Analysis of *Mn-HexA1* and *Mn-HexA2* in Hepatopancreas and Ovaries at Different Stages

**Figure 5:**
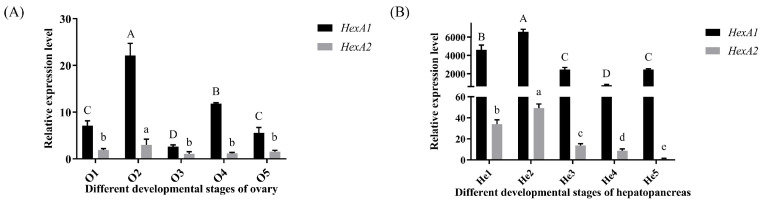
qPCR analysis of *Mn-HexA1* and *Mn-HexA2* expression patterns during distinct developmental stages in (**A**) ovarian and (**B**) hepatopancreatic tissues. Data are presented as mean ± SD (n = 6). Significant differential expression was observed between *Mn-HexA1* and *Mn-HexA2* across all developmental stages (*p* < 0.05). Uppercase letters indicate statistically significant differences among *Mn-HexA1* expression groups (*p* < 0.05), whereas lowercase letters denote significant variations among *Mn-HexA2* expression groups (*p* < 0.05). The developmental stages from O1-O5 and He1-He5 are defined in Table S1.

In ovarian tissues (Figure 5A), both *Mn-HexA1* and *Mn-HexA2* exhibited significant upregulation from stage O1 (undeveloped stage of Ovary) to O2 (developing stage of Ovary) (*p* < 0.05), reaching peak expression levels at O2. The expression of *Mn-HexA1* at O2 was significantly higher than all other developmental stages (*p* < 0.01), while its O3 (nearly ripe stage of the ovary) expression was markedly lower compared to other periods (*p* < 0.05). Similarly, *Mn-HexA2* demonstrated significantly elevated expression at O2 relative to other ovarian stages (*p* < 0.05), though no significant differences were observed among remaining stages. Notably, *Mn-HexA1* expression consistently surpassed *Mn-HexA2* levels across all ovarian developmental periods (*p* < 0.05).

In the hepatopancreas (Figure 5B), both *Mn-HexA1* and *Mn-HexA2* displayed a highly significant increase in expression from stage He1 (hepatopancreas in O1 stage) to He2 (hepatopancreas in O2 stage) (*p* < 0.01), reaching peak levels at He2 stage that were substantially higher than all other developmental stages (*p* < 0.01). In addition, *Mn-HexA1* expression at He4 (hepatopancreas in O4 stage) was significantly lower compared to other periods (*p* < 0.01), while *Mn-HexA2* exhibited its lowest expression at He5 (hepatopancreas in O5 stage) (*p* < 0.01). Furthermore, *Mn-HexA1* expression consistently and significantly exceeded *Mn-HexA2* levels across all hepatopancreatic developmental stages (*p* < 0.01).

### 2.4. Localization of Mn-HexA1 and Mn-HexA2 in Different Tissues

In situ *hybridization* (ISH) analysis demonstrated that both *Mn-HexA1* and *Mn-HexA2* exhibited strong hybridization signals in follicle cells (FCs), nurse cells (N), and the cytoplasmic membrane (CM) throughout all ovarian developmental stages. Notably, the hybridization signals of these two genes were generally weaker during the He2 stage (Figure S3).

### 2.5. Functional Analysis of Mn-HexA1 and Mn-HexA2

#### 2.5.1. Interference Efficiency

**Figure 6:**
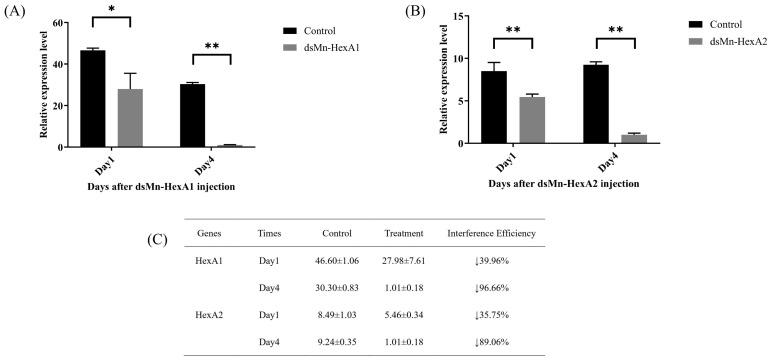
Expression analysis of (**A**) *Mn-HexA1* and (**B**) *Mn-HexA2* in *Macrobrachium nipponense* hepatopancreas following dsRNA injection, with (**C**) corresponding interference test data. Values represent mean ± SD (n = 6). Asterisks indicate statistical significance: * (*p* < 0.05), ** (*p* < 0.01). Downward arrows (↓) denote expression suppression.

To further investigate the roles of *Mn-HexA1* and *Mn-HexA2* in ovarian maturation, we performed *RNA interference* (RNAi) experiments. As demonstrated in Figure 6, the knockdown efficiency for *Mn-HexA1* reached 39.96% on day 1 and 96.66% on day 4 post-injection (*p* < 0.05). Similarly, *Mn-HexA2* showed knockdown efficiencies of 35.75% on day 1 and 89.06% on day 4 (*p* < 0.01), with both genes exhibiting statistically significant silencing effects.

#### 2.5.2. Effect of *Mn-HexA1* and *Mn-HexA2* Knockdown on Ovarian Development of *M. nipponense*

**Figure 7:**
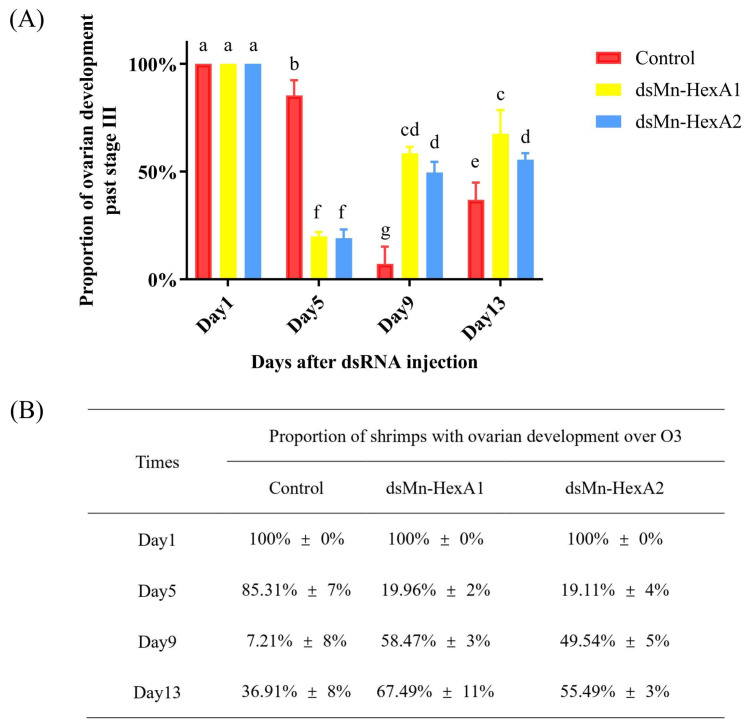
(**A**) The percentage of female *Macrobrachium nipponense* ovaries past stage III after injection of dsRNA, with (**B**) corresponding interference test data. Different letters indicate significant differences. *p* < 0.05 was considered to be statistically significant.

The comparative ovarian development between the experimental and control groups is presented in Figure 7. At trial initiation, all prawns were at O3. By day 5 after injection of dsRNA, less than 20% of shrimps in both experimental groups had ovarian development over O3, while over 80% entered the second reproductive cycle. In stark contrast, 85.31% of the control groups remained over O3 in the first cycle (*p* < 0.01), though no significant difference existed between the two experimental groups. On day 9, only 7.21% of prawns in control groups progressed over O3 versus approximately 50% in the two experimental groups (*p* < 0.01). Although *Mn-HexA1*-silenced groups showed marginally faster progression than *Mn-HexA2*-silenced group, the inter-group difference remained non-significant. On day 13, the proportion of shrimp ovaries over O3 in all groups gradually increased, with 36.91% in the control groups, compared to 67.49% and 55.49% in the *Mn-HexA1*-silenced and *Mn-HexA2*-silenced groups, respectively. There is still a highly significant difference between the control groups and the two experimental groups (*p* < 0.01). Notably, the *Mn-HexA1*-silenced group demonstrated significantly accelerated ovarian development relative to the *Mn-HexA2*-silenced group (*p* < 0.05).

## 3. Discussion

Hexosaminidases, as crucial members of glycosyl hydrolase family 20 (GH20) [17], exist in three primary isoforms: the major *HexA* and *HexB* isozymes, and the minor *HexS* variant. Structural studies reveal that *HexA* functions as a heterodimer of non-covalently linked α and β subunits, while *HexB* forms a homodimer of two β subunits, and HexS constitutes a homodimer of two α subunits [18]. Biochemically, their main functions include chitin degradation, protein N-glycan modification, glycoconjugates degradation, and sperm–egg recognition.

In humans, hexosaminidases is responsible for the hydrolysis of the terminal GalNAc residue from the GM2 ganglioside within the lysosome [19]. Specifically, mutations in the HEXA and HEXB genes lead to fatal neurodegenerative diseases: Tay–Sachs disease and Sandhoff disease, respectively [20,21,22]. Recent advances have revealed that a portion of cellular hexosaminidase activity may originate from extracellular vesicles (EVs) [23], while βGalNAc-Rhod-CM(Net_2_) has been identified as an effective molecular probe for monitoring hexosaminidase activity in live cells [24].

Altered hexosaminidase activity has been documented in various pathological conditions beyond lysosomal storage disorders, including several cancer types [25,26], asthma [27,28], and myeloproliferative disorders [29]. In addition, hexosanase is widely distributed in various organisms, such as plants [30,31], bacteria [32], and fungi [33].

In insects, emerging research has elucidated the functional roles of hexosaminidases, primarily involving two distinct mechanisms: modification of N-glycan structures in cellular glycoproteins [34] and chitin degradation during cuticle remodeling [35]. These enzymes exhibited complementary catalytic activities to chitinase: while chitinase cleaves internal bonds, hexosaminidases release terminal residues. Critical evidence comes from *RNA interference* studies demonstrating that hexosaminidase knockdown leads to lethal pupal entrapment within the exoskeleton [36]. This essential developmental function has positioned hexosaminidases as promising molecular targets for next-generation bioinsecticides [37].

The current understanding of hexosaminidase in crustaceans remains remarkably limited. To date, the only reported evidence comes from a transcriptomic study of *Palaemon gravieri* under saline stress conditions, where researchers identified putative HexA gene fragments but without subsequent functional validation [38]. This substantial knowledge gap highlights the need for systematic investigations into hexosaminidase’s physiological roles in crustacean species.

Extensive studies have demonstrated that hexosaminidase exhibits remarkable activity in spermatozoa and plays a crucial role in egg–sperm recognition [15]. Some researchers successfully purified and characterized *β-hexosaminidase* localized on the sperm membrane of *Drosophila melanogaster* [13]. Subsequent investigations confirmed its functional conservation with human orthologs, revealing direct involvement in sperm–zona pellucida binding and penetration in vitro [11]. In addition, hexosaminase can also modify the sperm receptor glycoprotein on the egg envelope, blocking polysperms and playing an important role in primary binding between gametes [14,15,16]. However, the potential regulatory effects of hexosaminidase on ovarian development remain unexplored, representing a significant knowledge gap in reproductive biology.

The current study identified *Mn-HexA1* and *Mn-HexA2* through comparative transcriptomic analysis of hepatopancreatic tissue at ovarian stages O1 and O2 in *M. nipponense*, with both isoforms showing significant upregulation during the transition from stage 1 to stage 2 [8]. KEGG enrichment reveals that the “lysosome” signaling pathway (map04142) is closely related to ovarian development, and *Mn-HexA1* and *Mn-HexA2* are located in this pathway. Several key lysosomal pathway components, *Cathepsin D* [8], *Cathepsin L* [39], and *Niemann–Pick C1 protein* [40] have been demonstrated to promote ovarian development, while lysosomal acid lipase plays a role in gonadal differentiation [41]. These findings collectively highlight the diverse functional roles of the lysosomal pathway in crustacean ovarian development.

Current evidence establishes that the lysosomal pathway containing HexA primarily facilitates macromolecule transport and catabolism. Of particular relevance to ovarian development, lysosomes directly participate in steroidogenesis through two key mechanisms: (1) generation of steroid precursors and (2) degradation of regulators governing ovarian steroid production, thereby maintaining cellular steroid homeostasis [42]. Lysosomes play a crucial role in steroidogenesis by releasing cholesterol from endocytosed LDL. This process involves lysosomal enzymes and cooperative action of NPC1 (a lysosomal membrane cholesterol transporter) and NPC2 (a soluble cholesterol-binding protein) [43]. Lysosomes also participate in degrading regulators of ovarian steroidogenesis, including LH-LHR complexes [44], FSH-FSHR complexes [45], and intrinsic PGF2α receptors [46]. Notably, our prior work confirmed that the lysosomal *LIPA* gene supports gonadal energy provision in *M. nipponense* through hydrolysis of triglycerides and cholesterol esters [41]. Some known functions of the *HexA* gene also focus on these aspects [16], so it may also participate in some part of the process, and the specific mechanism needs further study.

Bioinformatic analysis revealed that *Mn-HexA1* and *Mn-HexA2* share similar conserved domains and identical functional domains, suggesting overall functional similarity. However, sequence alignment showed limited homology at both nucleotide and amino acid levels, with only 58.43% amino acid identity. This substantial divergence indicates potential functional specialization between the two isoforms. Phylogenetic analysis provided further support for this hypothesis; although all identified orthologs from other species remain computationally predicted without experimental validation, the observation that *Mn-HexA1* and *Mn-HexA2* do not initially cluster together in the evolutionary tree reinforces the likelihood of functional divergence between *Mn-HexA1* and *Mn-HexA2*.

The expression profiling revealed striking differences between the two isoforms, with *Mn-HexA1* consistently exhibiting significantly higher expression levels than *Mn-HexA2* across all examined tissues and developmental stages. This pronounced disparity suggests that *Mn-HexA1* likely exerts more substantial physiological impacts on *M. nipponense* compared to its counterpart. The ubiquitous high expression of *Mn-HexA1* throughout multiple developmental phases implies its potential multifunctional roles, possibly extending beyond ovarian development to include stress responses such as salinity adaptation [38]. In contrast, the stage-restricted expression pattern of *Mn-HexA2* indicates a more specialized functional repertoire. The expression of *Mn-HexA1* in ZS, L15, and PL10 stages was significantly higher than that in other stages, and the expression level of *Mn-HexA2* did not fluctuate significantly in the early stage of embryonic development, while the expression of L15 and PL10 was significantly higher than that in other stages. ZS is the period when *M. nipponense* is about to emerge from the membrane, L15 is the critical period for metamorphosis and development of *M. nipponense*, and PL10 is the critical period for gonadal differentiation of *M. nipponense* [47]. These synchronized expression peaks during physiologically demanding developmental transitions strongly imply that *Mn-HexA1* and *Mn-HexA2* played a crucial role during the critical period. Based on the fundamental hydrolytic function of hexosaminidases, we speculate that *Mn-HexA1* and *Mn-HexA2* may be involved in energy regulation processes during these key developmental events.

Comparative analysis revealed significantly elevated expression of *Mn-HexA1* and *Mn-HexA2* across multiple tissues during the reproductive season compared to non-reproductive periods, suggesting enhanced metabolic activity potentially linked to gonadal development. Particularly noteworthy was the predominant expression in hepatopancreas, where transcript levels of *Mn-HexA1* and *Mn-HexA2* substantially exceeded those in other tissues (*p* < 0.01), indicating their crucial hepatic functions. Temporal expression profiling of ovarian and hepatopancreatic tissues demonstrated consistent upregulation of both *Mn-HexA1 and Mn-HexA2* from stage I (O1, He1) to stage II (O2, He2), peaking at stage II. This developmental window (O1–O2) corresponds to the critical vitellogenic phase in *M. nipponense*, during which the hepatopancreas supplies essential glycoproteins and energy metabolites for ovarian development [48]. This result once again proves that *Mn HexA* may be involved in the energy regulation process of ovarian development.

The *RNA interference* experiment, conducted for the first time in *M. nipponense* under natural pond conditions, yielded significant findings. All experimental specimens were synchronized at ovarian stage III prior to dsRNA administration. By day 5 post-interference, both *Mn-HexA1*-silenced and *Mn-HexA2*-silenced groups had transitioned to the second reproductive cycle, whereas control specimens took until day 9 to achieve comparable progression (Figure 7). Notably, the *Mn-HexA1* knockdown group exhibited progressively accelerated ovarian development compared to the *Mn-HexA2* knockdown group, with statistically significant differences emerging by day 13 (*p* < 0.05). It is concluded that both subtypes of *Mn-HexA* genes inhibit ovarian development, and the effect of *Mn-HexA1* is stronger than that of *Mn-HexA2*. This study represents the first functional characterization of *HexA* genes in crustacean ovarian development, providing novel insights into the molecular regulation of reproduction in *M. nipponense*. In the follow-up experiments, it is a good choice to enhance the effect of RNAi by feeding with feed, so as to explore more functions of the genes [49,50].

Current understanding of hexosaminidase’s reproductive functions remains remarkably limited, with existing studies primarily focused on protein purification [18]. As the first comprehensive study combining expression profiling and RNAi-based functional analysis of *hexosaminidases* in crustacean reproduction, our work provides significant mechanistic insights into this vital biological process.

Three key conclusions emerge with certainty: (1) Both *Mn-HexA1 and Mn-HexA2* serve as critical negative regulators of ovarian development in *M. nipponense*, representing only the second identified class of such inhibitory factors in this species [51]. This deepens the understanding of the mechanistic study of crustacean ovarian development. (2) This work provides the first experimental evidence that hexosaminidases functionally regulate ovarian development. (3) This work is the first to explore the function of hexosaminidase in crustaceans. Intriguingly, emerging research in humans demonstrates that hepatic *HexA* directly modulates insulin-like growth factor signaling and glucose transport [52], a mechanism strikingly consistent with our hypothesis of *Mn-HexA* involvement in energy regulation, but it still needs to be strictly verified through targeted experiments.

## 4. Materials and Methods

### 4.1. Experimental Animals and Breeding Conditions

Healthy female *Macrobrachium nipponense* specimens (ovarian stage III; mean body weight ±0.5 g) were obtained from the Freshwater Fisheries Research Center of the Chinese Academy of Fishery Sciences (Wuxi, Jiangsu Province, China). The prawns were cultured in outdoor earthen ponds (70 m × 25 m × 0.7 m) containing cylindrical net cages (0.8 m diameter × 1 m height) under natural conditions. The ponds were supplemented with *Hydrilla verticillata* vegetation to simulate natural habitats. The water temperature was maintained at 25–30 °C throughout the experimental period. The specimens were fed twice daily (morning and evening) with commercial feed at 5% of total body weight.

### 4.2. Tissue Sample Collection

Various tissues including the eyestalk, cerebral ganglion, heart, hepatopancreas, gill, muscle, and gonads were dissected from *M. nipponense* specimens and immediately flash-frozen in liquid nitrogen. All collected tissues were subsequently stored at −80 °C until further analysis. Additionally, whole-body samples from different embryonic developmental stages and larval phases were systematically collected and preserved under identical conditions (−80 °C). The specific classification criteria for different developmental stages of ovaries, the hepatopancreas, and embryonic developmental periods are summarized in Table S1 [8,53].

### 4.3. Genes Cloning

The total RNA of *M. nipponense* at different developmental stages and different tissues was extracted using RNAiso Easy reagents (TakaRa, Dalian, China) according to the manufacturer’s instructions, and quality was assessed by 1.2% agarose gel electrophoresis. According to the manufacturer’s instructions, we converted single-stranded RNA to single-stranded cDNA using the M-MLV reverse transcriptase kit (TaKaRa). Then, the synthesized cDNA was kept at −20 °C for subsequent quantitative real-time PCR (qPCR) reaction to detect the expression pattern of *the Mn-HexA1* and *Mn-HexA2* in *M. nipponense.* EIF was used as the internal reference gene; this has been proved before. The content of Mn-CH7D mRNA was calculated by the 2^−ΔΔCT^ method.

Total RNA was isolated from various tissues and developmental stages of *M. nipponense* using RNAiso Easy reagent (TakaRa) following the manufacturer’s protocol. RNA integrity was verified by 1.2% agarose gel electrophoresis. First-strand cDNA was synthesized from 1 μg of total RNA using the M-MLV Reverse Transcriptase Kit (Takara) according to the manufacturer’s instructions. The resulting cDNA products were stored at −20 °C for subsequent quantitative real-time PCR (qPCR) analysis.

The expression profiles of *Mn-HexA1* and *Mn-HexA2* in *M. nipponense* were analyzed by qPCR using EIF as the internal reference gene, as previously validated [54]. Relative mRNA levels were quantified using the 2^−ΔΔCT^ method [55].

### 4.4. Bioinformatics Analysis

The cDNA fragments of the target genes *Mn-HexA1* and *Mn-HexA2* were obtained from our laboratory’s established cDNA library, which was previously constructed from hepatopancreatic transcriptomes of *M. nipponense* at different ovarian developmental stages [7]. Amino acid sequence alignment was analyzed by DNAMAN 9.0 software. The phylogenetic tree was constructed based on *Mn-HexA1*, *Mn-HexA2,* and their orthologs in other species by the neighbor-joining (NJ) method using MEGA 11.0 software. Potential open reading frames were identified using the ORF Finder tool (https://www.ncbi.nlm.nih.gov/orffinder/, accessed on 1 July 2024). The functional domains within the protein sequences were identified using the Conserved Domain Database (CDD) search tool available through NCBI (https://www.ncbi.nlm.nih.gov/Structure/cdd/wrpsb.cgi, accessed on 2 July 2024). Transmembrane helices in the amino acid sequences were predicted using DeepTMHMM, a deep-learning-based method for transmembrane topology prediction (https://dtu.biolib.com/DeepTMHMM, accessed on 2 July 2024). Protein functional domains were predicted using InterPro (https://www.ebi.ac.uk/interpro/, accessed on 2 July 2024). Signal peptide prediction was performed using SignalP-6.0 (https://services.healthtech.dtu.dk/services/SignalP-6.0/, accessed on 2 July 2024). Protein physicochemical characteristics including molecular weight (MW), isoelectric point (pI), and amino acid composition were calculated using ProtParam tool available on ExPASy server (https://web.expasy.org/protparam/, accessed on 2 July 2024). The three-dimensional protein structures were predicted using the SWISS-MODEL homology modeling server (https://swissmodel.expasy.org/, accessed on 3 July 2024) and subsequently analyzed with ESPript (https://espript.ibcp.fr/ESPript/cgi-bin/ESPript.cgi , accessed on 3 July 2024). Gene-specific primers were designed using the NCBI Primer-BLAST tool (https://www.ncbi.nlm.nih.gov/tools/primer-blast/, accessed on 3 July 2024). The primers used in this study are listed in Table S2.

### 4.5. In Situ Hybridization

Ovarian samples (stages 1–5) and stage 2 hepatopancreatic tissues fixed in 4% paraformaldehyde solution were used for ISH studies. DIG-labeled antisense and sense RNA probes were designed based on *Mn-HexA1* and *Mn-HexA2* cDNA sequences using Primer5 software. The experimental procedures followed previously established protocols [56]. HE groups represent the blank control of routine hematoxylin–eosin staining, negative indicates the control group hybridized with sense probe, and positive indicates the experimental group hybridized with antisense probe. The probe sequences are shown in Table S2.

### 4.6. RNAi Experiment

The primers for double-stranded RNA (dsRNA) synthesis were designed using SnapDragon online software (https://www.flyrnai.org/cgi-bin/RNAi_find_primers.pl , accessed on 5 July 2024). The dsRNAs targeting *Mn-HexA1* and *Mn-HexA2* were synthesized using the TranscriptAid^™^ T7 High Yield Transcription Kit (Thermo Fisher Scientific, Waltham, MA, USA), with specific primers listed in Table S2. The concentration of synthesized dsRNA was measured at 260 nm using a BioPhotometer (Eppendorf, Hamburg, Germany), with purity verified by A260/A280 ratio (1.8–2.0).

To evaluate the interference efficiency, a short-term *RNA interference* test was conducted separately, and samples of hepatopancreas were collected on the 1st and 4th day after injection of dsRNA. After total RNA was extracted and reverse-transcribed into cDNA, the expression level of the target gene was detected by qPCR to determine the interference efficiency. Total RNA was extracted from collected samples and reverse-transcribed into cDNA. The knockdown efficiency was quantitatively assessed by measuring target gene expression levels using qPCR.

A long-term *RNA interference* experiment was conducted to randomly allocate 270 healthy female *M. nipponense* (1.46 ± 0.19 g) from phase 3 to 9 aquaculture ponds in equal proportions, namely the HexA1 experimental group, HexA2 experimental group, and control group, with 3 parallel groups in each group (n = 30). The experimental groups was injected with dsRNA at a dose of 4 μg/g (calculated per gram of body weight) through the pericardial cavity of *M. nipponense*, while the control groups were injected with the same unit dose of *dsGFP*. The injection frequency was once every four days. The ovarian development stages of each shrimp were observed and recorded every day, and the proportion of shrimps whose ovarian development exceeds the third stage was determined.

### 4.7. Data Analysis

All quantitative data are presented as mean ± standard deviation (mean ± SD). Statistical analyses were performed using SPSS Statistics 24.0 (IBM, Armonk, NY, USA). One-way analysis of variance (ANOVA) followed by Duncan’s multiple comparison test was employed to determine significant differences between control and treatment groups. Differences were considered statistically significant at *p* < 0.05 and highly significant at *p* < 0.01. Relative gene expression levels were calculated using the comparative 2^−ΔΔCT^ method.

## 5. Conclusions

This study is the first discovery of gonadal development function of *β-hexosaminidase A* in crustaceans. The RNAi test of *Macrobrachium nipponense* was carried out in a mud pond for the first time. The synchronous interference test of two subtypes of the same gene in *M. nipponense* was carried out for the first time. It is also the second ovarian development inhibitory gene found in *M. nipponense*. This study provides the first comprehensive functional characterization of *Mn-HexA1* and *Mn-HexA2* in crustacean reproduction through integrated bioinformatic, molecular, and physiological approaches, confirming the inhibitory effect of *Mn HexA* on ovarian development. It also provides some new insights and ideas to solve the production problem of rapid sexual maturation and advance our understanding of crustacean reproductive endocrinology while identifying potential molecular targets for aquaculture management.

## Data Availability

The data presented in this study are available on request from the corresponding author for scientific purposes.

## Supplementary Materials

The following supporting information can be downloaded at: https://www.mdpi.com/article/10.3390/ijms26125459/s1.
