# Supplementary material for: Functional Characterization of Two β-Hexosaminidase A Isoforms During Ovarian Development in Macrobrachium nipponense
Source: Int J Mol Sci. 2025 Jun 6;26(12):5459. doi: 10.3390/ijms26125459 (PMC12192582; doi:10.3390/ijms26125459)
Supplement: Supplementary file 1 [file ijms-26-05459-s001.zip › Table S1.pdf]

Table S1. Specific classification criteria of ovary, hepatopancreas and embryonic development stages of *Macrobrachium nipponense*.

Stages	Characteristic
O1	oocyte stage, undeveloped stage, transparent, oocyte proliferation
O2	primary vitellogenesis stage, developing stage, yellow or khaki, original age of yolk
O3	secondary vitellogenesis stage, nearly-ripe stage, light green, secondary yolk
O4	ripe stage, dark green, yolk termination
O5	emptying stage, gray, recession
He1-He5	hepatopancreas corresponding to O1-O5 at the same stage
CS	cleavage stage, cell cleavage
BS	blastula stage, appearance of yolk sack
GS	gastrula stage, transparent area occurred on the end of embryo
NS1	the early stage of nauplius stage, no visible somites and formation of primordium
NS2	the late stage of nauplius stage, occurrence of somites and formation of primordium
PS	protozoea stage, occurrence of eye pigments
ZS	zoea stage, formation of cephalothorax, appendage and interior structure
L1	the 1st-day larvae after hatching from the embryonic membrane
L5	the 5th-day larvae after hatching from the embryonic membrane
L10	the 10th-day larvae after hatching from the embryonic membrane
L15	the 15th-day larvae after hatching from the embryonic membrane
PL1	the 1st day after metamorphosis
PL5	the 5th day after metamorphosis
PL10	the 10th day after metamorphosis
PL15	the 15th day after metamorphosis
PL20	the 20th day after metamorphosis
PL25	the 25th day after metamorphosis
