# Supplementary material for: Functional Characterization of Two β-Hexosaminidase A Isoforms During Ovarian Development in Macrobrachium nipponense
Source: Int J Mol Sci. 2025 Jun 6;26(12):5459. doi: 10.3390/ijms26125459 (PMC12192582; doi:10.3390/ijms26125459)
Supplement: Supplementary file 1 [file ijms-26-05459-s001.zip › Table S2.pdf]

Table S2. The primers used in this study.

Primer	Primer Sequence (5′-3′)
Mn-HexA1 F1 (ORF)	GATGGCTCCGAAGCACAAAAAC
Mn-HexA1 R1 (ORF)	GCTCTACTCTGAAGCCTCTGTC
Mn-HexA1 F2 (ORF)	CTGACAGACCATCTGATGATGG
Mn-HexA1 R2 (ORF)	TATCGTTTTGTAACCTGCCTGC
Mn-HexA2 F (ORF)	CCTGCATCTGGGACCATCATAG
Mn-HexA2 R (ORF)	AGATGTGGATGATGGTGTCGTC
Mn-HexA1 F (qPCR, ORF)	AGGTTAAAAACAGCAATGTTCG
Mn-HexA1 R (qPCR, ORF)	CCATCATCAGATGGTCTGTCAG
Mn-HexA2 F (qPCR, ORF)	GGCTTTCATTTTCTTTTCCGGC
Mn-HexA2 R (qPCR, ORF)	CTATGATGGTCCCAGATGCAGG
EIF F (qPCR)	CATGGATGTACCTGTGGTGAAAC
EIF R (qPCR)	CTGTCAGCAGAAGGTCCTCATTA
Mn-HexA1 anti-probe (ISH)	5'- AACTCCACAGTTTTTCGGCAACCACTGAA-3'
Mn-HexA1 probe (ISH)	5'- TTCAGTGGTTGCCGAAAAACTGTGGAGTT-3'
Mn-HexA2 anti-probe (ISH)	5'- ATGAAAGCCTGACAGGTTTTCCTCATTATACTTG-3'
Mn-HexA2 probe (ISH)	5'- CAAGTATAATGAGGAAAACCTGTCAGGCTTTCAT-3'
ds*Mn-HexA1* F (RNAi)	TAATACGACTCACTATAGGG GGTGACGAGGTCAGCTTTTC
ds*Mn-HexA1* R (RNAi)	TAATACGACTCACTATAGGG TCAGCTGTTTTTGTGCTTCG
ds*Mn-HexA2* F (RNAi)	TAATACGACTCACTATAGGG TCTATGGCGGTGAACCTCTC
ds*Mn-HexA2* R (RNAi)	TAATACGACTCACTATAGGG TCTCCAGCTGCTTTCAGGAT
ds*GFP* F (RNAi)	TAATACGACTCACTATAGGG ACGAAGACCTTGCTTCTGAAG
ds*GFP* R (RNAi)	TAATACGACTCACTATAGGG AAAGGGCAGATTGTGTGGAC
