# Supplementary figures and images for: Functional Characterization of Two β-Hexosaminidase A Isoforms During Ovarian Development in Macrobrachium nipponense
Source: Int J Mol Sci. 2025 Jun 6;26(12):5459. doi: 10.3390/ijms26125459 (PMC12192582; doi:10.3390/ijms26125459)

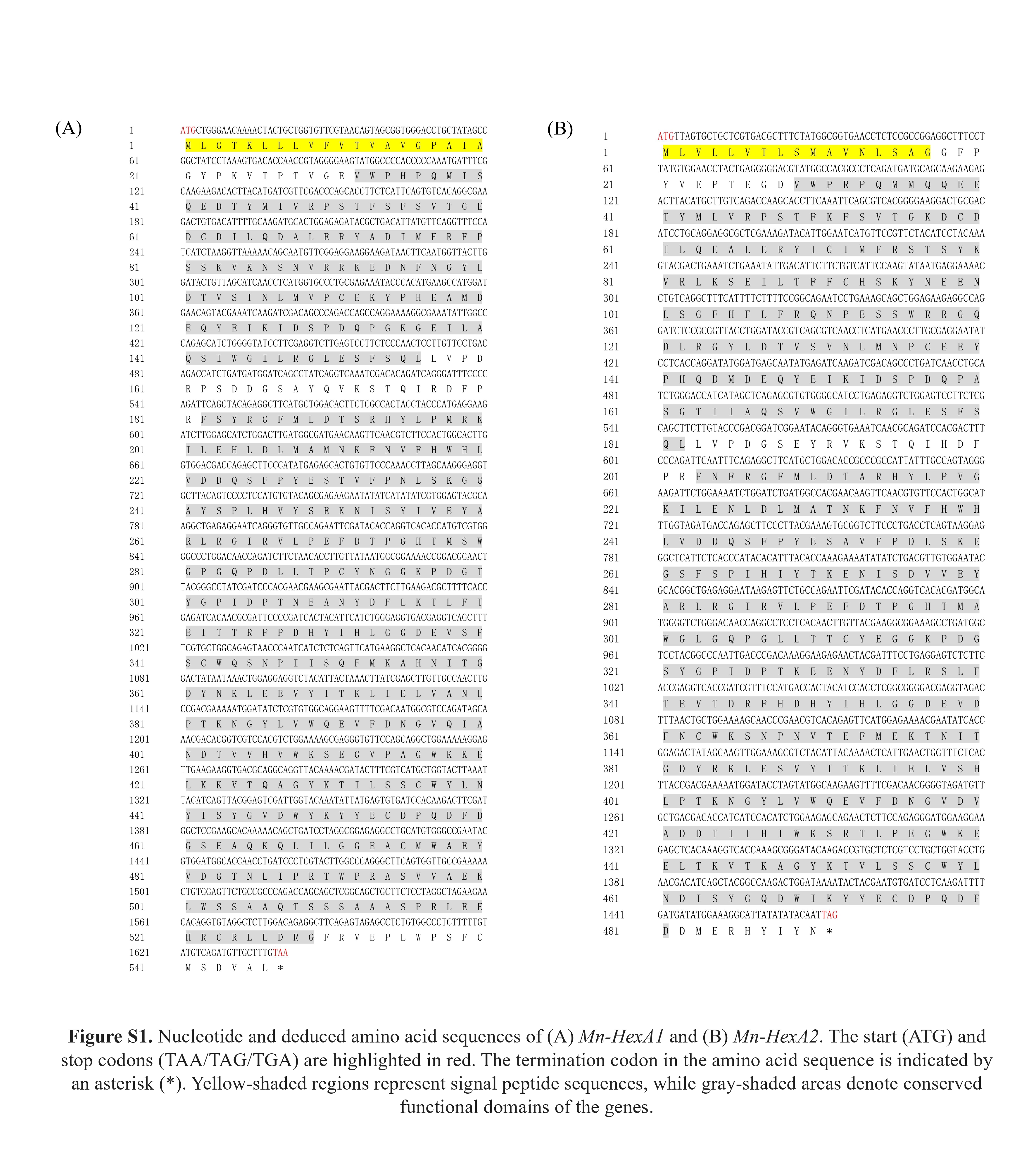

Supplement: Supplementary file 1 [file ijms-26-05459-s001.zip › Figure S1.jpg]

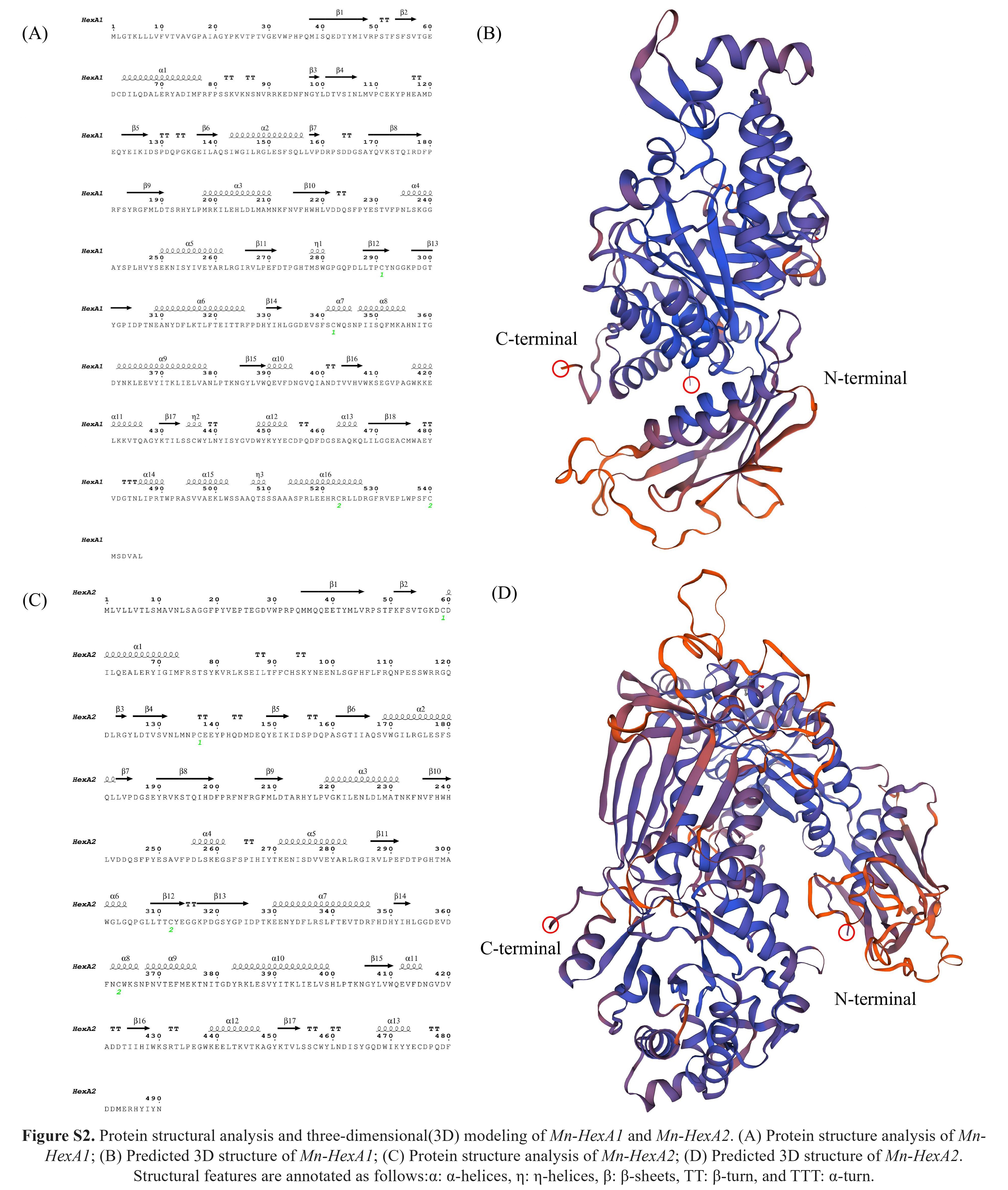

Supplement: Supplementary file 1 [file ijms-26-05459-s001.zip › Figure S2.jpg]

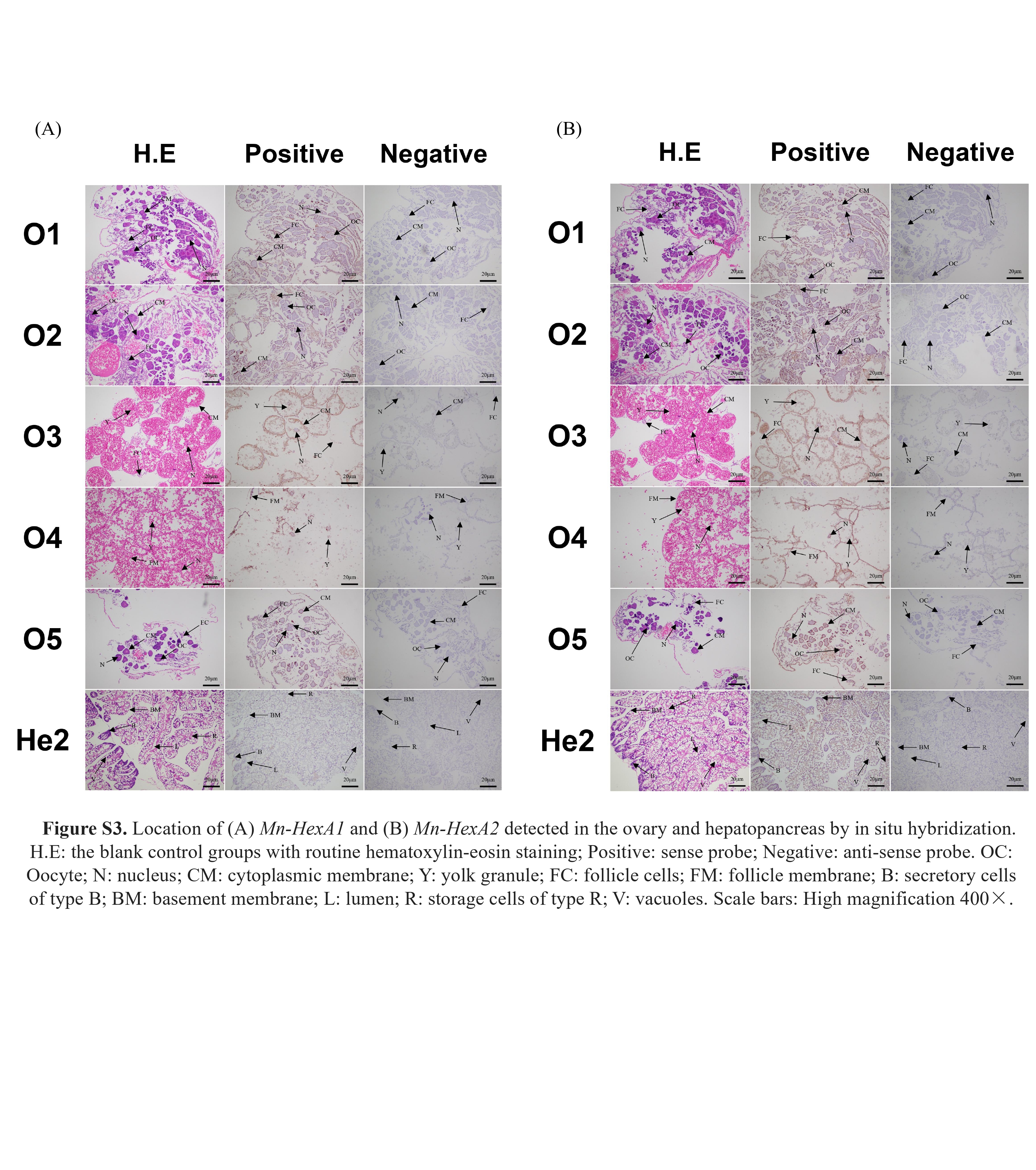

Supplement: Supplementary file 1 [file ijms-26-05459-s001.zip › Figure S3.jpg]
